# Self-compassion and grit mediated the relation between mindfulness and mind wandering based on cross-sectional survey data

**DOI:** 10.1038/s41598-024-58395-y

**Published:** 2024-04-20

**Authors:** Rebecca Y. M. Cheung, Lemuela Djekou

**Affiliations:** School of Psychology and Clinical Language Sciences, University of Reading, Reading, UK

**Keywords:** Grit, Meditators, Mindfulness, Mind wandering, Self-compassion, Psychology, Signs and symptoms

## Abstract

Previous research suggests that mindfulness and mind wandering are opposing constructs. However, little is known about why and how they are negatively related. Through a process-oriented approach, this cross-sectional study tested self-compassion and grit as mediators for the relation between mindfulness and mind wandering. A total of 487 self-identified meditators were recruited from the UK (241 female, 49.49%). Participants reported a mean age of 38.98 years (*SD* = 10.03), with an average of 2.26 h of meditation practice per week (*SD* = 4.47). Upon informed consent, the participants completed a self-report questionnaire that assessed the core variables under study. Path analysis indicated that mindfulness was related to self-compassion. Greater self-compassion was, in turn, related to greater grit, which was then related to lower mind wandering. Bootstrapping analysis further indicated that self-compassion and grit as mediators between mindfulness and mind wandering, above and beyond age, gender, hours of meditation, income, and education as covariates. The cross-sectional findings provided initial evidence of mediation by showing that mindfulness and mind wandering were related through self-compassion and grit.

## Introduction

Mindfulness refers to the awareness arising from paying attention to the present moment, on purpose, and without judgment^1^. Over the last decade, a growing body of research has indicated that mindfulness is associated with better psychological functioning, including better emotion regulation^2,3^, better life satisfaction^4,5^, and fewer affective symptoms^6,7^. Of note, the relation between the opposing constructs of mindfulness and mind wandering has received some scientific attention^8^. According to Smallwood and Schooler^9^, mind wandering refers to task-unrelated thoughts and are stimulus-independent. When individuals are dispositionally more mindful, they are also less likely to engage in mind wandering^8^. This relation is further supported by mindfulness-based interventions involving university students, which show that mindfulness practice is related to fewer distracting thoughts and fewer episodes of mind wandering^8,10,11^. Despite the findings between the constructs, little is known about why and how mindfulness is associated with mind wandering. In addition, the studies in this area often involve university students^8,11^, rather than individuals from other developmental periods or other contexts. To address the gap in the literature, this cross-sectional study explored self-compassion and grit as the potential mediators between mindfulness and mind wandering in a sample of meditators residing in the UK.

### Mindfulness and self-compassion

As a correlate of mindfulness^12^, self-compassion may serve as a process between mindfulness and mind wandering. According to Goetz et al.^13^^, p. 351^, compassion refers to “the feeling that arises in witnessing another’s suffering and that motivates a subsequent desire to help”. Other scholars similarly define compassion as a response to suffering, which is universal among all human beings^14,15^. Gu et al.^16^ pointed out that compassion can be directed to the self or to other people. Importantly, self-compassion has three components including self-kindness, common humanity, and mindfulness during the encounter with suffering, as opposed to, self-judgment, isolation, and over-identification to specific experiences^15^. Although mindfulness is a component of self-compassion, Neff^15^ suggested that mindfulness forms the basis for self-kindness and common humanity, and that mindfulness and self-compassion are theoretically overlapping but distinct concepts^17^. Similarly, Voci et al.^12^ argued that self-compassion is an “heartful” aspect of mindfulness that highlights a caring attitude and heartfulness towards oneself.

Previous studies indicated that mindfulness, self-compassion, and mental health are closely connected^12,18,19^. Grounded in mindfulness-to-meaning theory^20^, mindfulness fosters people’s tendency towards decentering, i.e., to be aware of thoughts and feelings from a distance and examine them, instead of viewing them as necessarily true^21^. Rather than being on autopilot and habitually reacting to stressful situations, decentering further supports people to pause, have a broadened awareness and attention to the context, and reappraise the situation in a more objective and positive way^20^. Through this process, people have a greater capability to respond adaptively and skillfully to the stressor^20,21^, e.g., to generate compassion rather than criticisms towards the self and the others, which is further linked to a greater meaning in life. Supporting the basis of mindfulness-to-meaning theory^20^, previous research indicated that self-compassion mediated the relation between dispositional mindfulness and a myriad of psychological outcomes, including better psychological well-being among meditators and non-meditators^12,22^, greater academic resilience among underprivileged university students^23^, and lower severity of social anxiety among individuals with social anxiety disorder^24^.

### Mindfulness and grit

As discussed earlier, mindfulness-to-meaning theory^20^ postulates that by attending to the present moment without judgment, individuals are supported to broaden their awareness to information that may otherwise have gone unnoticed^25^. The broadened, non-judgmental, and present-moment awareness may serve as a foundation for skillful responses, as opposed to habitual patterns of autopilot, reactivity, or dysregulation. As such, mindfulness supports individuals’ awareness of conflicting goals and autonomy to prioritize long-term goals over hedonically pleasant activities^26^. This is consistent with the principles of self-determination theory^27^, which identifies autonomy as a fundamental psychological need.

When people are high on non-judgmental awareness and autonomous response to their needs, they may also have greater grit^27^. Duckworth et al.^28^ defined grit as people’s consistency of interests and perseverance of effort, i.e., the passion and tenacity in pursuing certain goals. Supporting the mindfulness-to-meaning theory [× 20] and self-determination theory^27^, recent studies suggested that mindfulness is associated with grit^29–31^. For instance, mindfulness facets including acting with awareness and non-judging were found to predict grit longitudinally^31^. In another study, mindfulness was associated with greater grit among athletes^30^. Specifically, athletes who were more mindful might be better at evaluating their setbacks non-judgmentally and regulating themselves, resulting in greater grit. In addition, gritty people are likely to persevere from setbacks and remain committed to their set goals^32^. As participants of mindfulness-based cognitive therapy, university students also reported experiencing greater grit at post-intervention^33^. Therefore, grounded in theories^20,27^ and the empirical literature^30,31^, the positive link between mindfulness and grit has been well-established.

### Self-compassion, grit, and mind wandering

According to Neff^15^, self-compassion allows individuals to be more understanding of their own setbacks as part of the common humanity in pursuing long-term goals. As such, self-compassion fosters a sense of relatedness to others, another innate psychological need outlined in self-determination theory^27^. Such relatedness and belongingness, according to Deci and Ryan^27^, is central for commitment and sustained motivation in pursuing goals. In addition to relatedness, the tendency to be self-compassionate also lowers self-judgement^34^ and the fear of failure^35,36^. Therefore, self-compassion may be crucial for enhancing grit^30^.

Self-compassion is also related to lower mind wandering among mildly to severely depressed individuals^37^. As discussed at the beginning of the introduction, mind wandering refers to thoughts that are unrelated to the task at hand and are independent from an existing stimulus^9^. When people have greater self-compassion, they are more likely to be gentle with themselves and focus on the tasks at hand, rather than wandering off to interfering thoughts such as criticism, rumination, and worries^38^. In Greenberg et al.’s study^37^, self-compassion further protected individuals from the damaging effects of maladaptive mind wandering on depressive symptoms. As such, self-compassion is central to better mental health outcomes, including greater grit, lower mind wandering, and fewer depressive symptoms.

### Grit and mind wandering

According to the optimal performance and health model of grit^39^, people’s grit, as indexed by their consistency of interests and perseverance of effort, motivates their self-regulation, thereby enhancing their well-being and achievement outcomes. Previous studies have shown that individuals with greater grit are less likely to procrastinate, have better self-regulation, and are more likely to maintain an interest in what they are doing, which allow them to perform better, remain diligent, and have sufficient discipline to achieve their long-term goals^40–42^. As such, grit and mind wandering may be inversely related. When people recognize their mind is wandering, a redirection of their thoughts to the present is crucial^43^, such that they can return to their task at hand. Khoo and Yang^44^ found that among gritty individuals, inhibitory control increased their resilience to ignore distracting stimuli such as smartphones. More specifically, they were more likely than were less gritty people to actively suppress distractions and remain focused on things that were relevant to their goal. Supporting the optimal performance and health model of grit^39^, the grittier the individual, the greater the ability to retain concentration and perseverance. This may further reduce the likelihood of mind wandering.

### The present study

**Figure 1:**
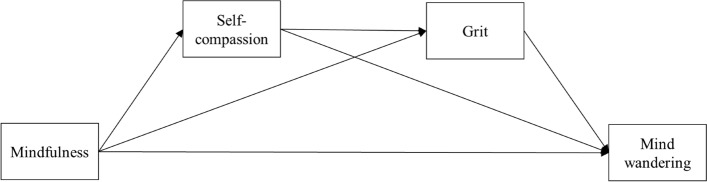
Conceptual model of grit and self-compassion as mediators between mindfulness and mind-wandering.

Grounded in existing theories and empirical findings^8,12,27,39^, a conceptual model was developed in Fig. 1. To test the conceptual model, the present study evaluated self-compassion and grit as mediators between mindfulness and mind wandering. Through cross-sectional data, it was hypothesized that self-compassion and grit would mediate the relation between mindfulness and mind wandering. Specifically, mindfulness was hypothesized to be positively related to self-compassion and grit, and negatively related to mind wandering. Self-compassion was hypothesized to be positively related to greater grit and negatively related to mind wandering. Grit was further hypothesized to be negatively related to mind wandering. Moreover, self-compassion and grit were hypothesized to mediate the relation between mindfulness and mind wandering. Given that the hours of meditation, age, gender, and socioeconomic status were found to be associated with the variables under study^45–50^, they were included as covariates in the hypothesized mediation model.

## Methods

### Participants

Assuming alpha = 0.05, *df* = 8, power = 0.8, null RMSEA = 0.10, and alternative RMSEA = 0.05, the minimum sample required for the path analysis under study was 383^51,52^. Conservatively guarding against outliers and invalid data, a larger sample of 487 meditation practitioners were recruited. Participants ranged from 19 to 60 years in age (*M* = 38.98 years; *SD* = 10.04; 50.21% women) and were recruited in the UK via Prolific (www.prolific.co). Inclusion criteria include (a) self-identified meditators, (b) current residence in the UK, (c) above 18 years of age, and (d) proficiency in English. To compensate for their time, the participants were given £4.55 as an incentive. Participants had an average of 37.97 months of meditation practice (*SD* = 61.82; median = 24; range 0–480), with an average of 2.26 h of meditation per week (*SD* = 4.46; median = 1.00; range 0 60). Given “hours of meditation per week” was skewed at 8.86 with a kurtosis at 96.39, the variable was ln-transformed for further analyses. Details of the demographic information are presented in Table 1.

**Table 1:** Demographic information of the sample (N = 487).

	M (SD)	N (%)
Gender		
Women		243 (49.90%)
Men		241 (49.48%)
Other		3 (0.62%)
Age (in years)	38.98 (10.03)	
Marital status		
Single		211 (43.33%)
Married		158 (32.44%)
Domestic partnership		90 (18.48%)
Divorced		18 (3.70%)
Separated		8 (1.64%)
Widowed		2 (0.41%)
Race		
Asian		13 (2.67%)
Black		15 (3.08%)
Mixed		14 (2.87%)
White		445 (91.38%)
Education		
Some high school		10 (2.05%)
High school diploma or equivalent		124 (25.47%)
Technical or vocational training		59 (12.11%)
Associate degree		15 (3.08%)
University degree		184 (37.78%)
Postgraduate degree		95 (19.51%)
Annual household income		
£0–5000		9 (1.85%)
£5001–15,000		53 (10.88%)
£15,001–25,000		77 (15.81%)
£25,001–35,000		89 (18.28%)
£35,001–45,000		62 (12.73%)
£45,001–55,000		52 (10.68%)
£55,001–65,000		30 (6.16%)
£65,001–75,000		40 (8.21%)
£75,001–85,000		21 (4.31%)
£85,001–95,000		18 (3.70%)
£95,001–105,000		15 (3.08%)
> £105,001		21 (4.31%)
Religion		
Agnostic		144 (29.57%)
Atheist		165 (33.88%)
Buddhist		10 (2.05%)
Catholic		45 (9.24%)
Christian		81 (16.63%)
Hindu		4 (0.82%)
Muslim		5 (1.03%)
No religion		28 (5.75%)
Other		5 (1.03%)
Experience in meditation (in months)	37.97 (61.82)	
Meditation per week (in hours)	2.26 (4.46)	
Meditation techniques or traditions		
Christian		7 (1.44%)
Islamic		2 (0.41%)
Samatha		2 (0.41%)
Secular		242 (49.69%)
Transcendental/mantra		21 (4.31%)
Vipassana		14 (2.87%)
Zen		99 (20.33%)
Multiple		35 (7.19%)
Others		65 (13.35%)

### Measures

#### Mindfulness

The 39-item Five Facet Mindfulness Questionnaire (FFMQ^53^) was used to assess mindfulness on facets consisting of five subscales including observing, acting with awareness, non-judging of inner experience, non-reactivity to inner experience, and describing. Participants rated on a 5-point Likert-type scale from 1 (never or very rarely) to 5 (very often or always true). Sample items included, “I pay attention to sensations, such as the wind in my hair or sun on my face” (observing), “I tell myself that I shouldn’t be feeling the way that I’m feeling” (non-judging of inner experience), “I rush through activities without being really attentive to them” (acting with awareness), “I perceive my feelings and emotions without having to react to them” (non-reactivity to inner experience) and “I can easily put my beliefs, opinions and expectations into words” (describing). Negatively worded items were reverse scored. The raw scores were then averaged, such that higher averaged scores indicated greater mindfulness. In this study, the Cronbach’s alpha = 0.94 and McDonald’s omega = 0.94 for this study.

#### Self-compassion

The 12-item Self-Compassion Scale Short Form (SCS-SF^54^) was used to assess self-compassion in participants on 6 subscales including, self-kindness, self-judgment, common humanity, isolation, mindfulness, and over-identification. Sample items included, “when I’m going through a very hard time, I give myself the caring and tenderness I need” (self-kindness), “judgemental about my own flaws” (self-judgment), “I try to see my failings as part of the human condition” (common humanity), “when I fail at something that’s important to me, I tend to feel alone in my failure” (isolation), “when something painful happens, I try to take a balanced view of the situation” (mindfulness) and “when I’m feeling down I tend to obsess and fixate on everything that’s wrong” (over-identification). Participants rated on a 5-point scale ranging from 1 (almost never) to 5 (almost always). Negatively worded items were reverse scored. The raw scores were then averaged, such that higher mean score indicated a greater self-compassion. In this study, the Cronbach’s alpha = 0.91 and McDonald’s omega = 0.91 for this study.

#### Grit

The 8-item Short Grit Scale (Grit-S^55^) was used to assess trait-level perseverance consisting of two subscales: Consistency of Interest and Perseverance of Effort. Participants rated the items on a 5-point scale from 1 (not at all like me) to 5 (very much like me). Sample items included, “I finish whatever I begin” (perseverance of interest) and “Setbacks don’t discourage me” (consistency of interest). Negatively worded items were reverse scored. The raw scores were then averaged, such that higher scores indicated greater grit. In this study, the Cronbach’s alpha = 0.87 and McDonald’s omega = 0.87 for this study.

#### Mind wandering

Mind wandering in participants were assessed via the 15-item Mind excessively wandering scale (MEWS^56^). A sample item included, “I can only focus my thoughts on one thing at a time with considerable effort”. Participants rated on a 4-point Likert scale from 0 (not at all or rarely) to 3 (nearly all of the time or constantly). Although the measure was initially developed for adults with attention deficit hyperactivity disorder (ADHD^56^), Mowlem et al.^57^ demonstrated measurement invariance between individuals with or without ADHD diagnosis. As such, it is valid for use among samples with or without ADHD. The raw scores were summed, such that higher summed scores indicated higher levels of mind wandering. In this study, the Cronbach’s alpha = 0.94 and McDonald’s omega = 0.95 for this study.

### Data analyses

Preliminary analyses including descriptive statistics and correlations were conducted using IBM SPSS Statistics, version 27. Path analysis was then conducted using MPLUS, version 8.3^58^ to investigate the hypothesized model, with self-compassion and grit as mediators for the relation between mindfulness and mind wandering. To examine the goodness-of-fit of the model to the data, Comparative fit index (CFI), Tucker-Lewis index (TLI), standardized root mean square residual (SRMR), and root mean squared error of approximation (RMSEA) were applied to assess the model’s goodness of fit. According to Kline^59^ and MacCallum et al.^51^, CFI and TLI values greater than 0.90 and SRMR and RMSEA values less than 0.10 suggest acceptable fit between the observed data and the hypothesized mediation model, whereas CFI and TLI values greater than 0.95 and SRMR and RMSEA values less than 0.05 suggest good fit between the observed data and the hypothesized mediation model. Bootstrapping was used to evaluate the mediating roles of self-compassion and grit, as this approach yields more precise estimates of the standard errors for the indirect effect compared to alternative approaches^60^. This dataset had no missing data. As the data did not consist of any outliers, all of the data were retained for analyses.

### Ethics approval

The present study was approved by ethics committee at The Education University of Hong Kong (REF: 2021-2022-0312) and was conducted in accordance with the ethical standards in the 1964 Declaration of Helsinki and its later amendments.

### Consent

Prior to the study administration, informed consent was obtained from all participants.

## Results

Table 2 shows the zero-order correlations, means, and standard deviations of the variables. Specifically, the core variables under study were related at *p*s < 0.001. As for the hypothesized mediation analysis, the path model fit adequately to the data, *χ*^*2*^(8) = 25.54, *p* = 0.001, CFI = 0.98, TLI = 0.94, RMSEA = 0.07; SRMR = 0.03 (see Table 3 for details). After controlling for hours of meditation per week, age, gender, income, and education, mindfulness was positively associated with self-compassion (β = 0.68, *p* < 0.001) and grit (β = 0.39, *p* < 0.001), and negatively associated with mind wandering (β =  − 0.31, *p* < 0.001). Self-compassion was, in turn, positively associated with grit (β = 0.22, *p* < 0.001) and negatively associated with mind wandering (β =  − 0.32, *p* < 0.001). Grit was also negatively related to mind wandering (β =  − 0.24, *p* < 0.001). Regarding the covariates, hours of meditation per week was positively related to mindfulness (*r* = 0.18, *p* = 0.001), self-compassion (β = 0.10, *p* = 0.01), and mind wandering (β = 0.13, *p* = 0.001). Age was positively related to mindfulness (*r* = 0.18, *p* < 0.001) and grit (β = 0.10, *p* = 0.02). Income was positively related to mindfulness (*r* = 0.14, *p* = 0.01), self-compassion (β = 0.09, *p* = 0.01), and grit (β = 0.08, *p* = 0.046). Gender and education were not related to the variables under study, *p*s > 0.05. Overall, the independent variables explained 54.4% of the variance for self-compassion (*R*^2^ = 0.54), 38.1% of the variance for grit (*R*^2^ = 0.38), and 56.3% of the variance for mind wandering (*R*^2^ = 0.56).

**Table 2:** Zero-order correlations, means, and standard deviations of the variables under study (*N* = 487).

Variable	(1)	(2)	(3)	(4)	(5)	(6)	(7)	(8)	(9)
(1) Age	–								
(2) Gender (0 = male, 1 = female)	0.053	–							
(3) Hours of meditation per week^†^	0.10	− 0.20**	–						
(4) Income	− 0.03	0.02	− 0.08	–					
(5) Education	− 0.04	− 0.001	− 0.10*	0.18***	–				
(6) Mindfulness	0.17***	− 0.05	0.16***	0.12*	0.01	–			
(7) Self-compassion	0.19***	− 0.05	0.22***	0.17**	0.03	0.72***	–		
(8) Grit	0.20***	0.01	0.12*	0.18**	0.10	0.57***	0.53***	–	
(9) Mind-wandering	− 0.20***	− 0.03	− 0.02	− 0.13*	− 0.04	− 0.66***	− 0.65***	− 0.58***	–
*M*	38.98	–	0.52	5.42	5.08	3.26	3.03	3.25	31.15
*SD*	10.03	–	0.83	2.80	1.58	0.52	0.77	0.77	9.73

Participants’ education ranged from 1 to 6: 1 = Some high school; 2 = High school/College graduate; 3 = Technical/Vocational training; 4 = Associate degree; 5 = University degree; 6 = Postgraduate degree. Monthly household income ranged from 1 to 12: 1 = £0–5000; 2 = £5001–15,000; 3 = £15,001–25,000; 4 = £25,001–35,000; 5 = £35,001–45,000; 6 = £45,001–55,000; 7 = £55,001–65,000; 8 = £65,001–75,000; 9 = £75,001–85,000; 10 = £85,001–95,000; 11 = £95,001–105,000; 12 ≥ £105,001.

*p < 0.05, **p < 0.01, ***p < 0.001.

^†^Participants’ hours of meditation per week was ln-transformed to minimize the skewness of the data for analyses.

**Table 3:** Parameter estimates and standard errors of the path model (N = 487).

Parameter	Unstandardized B (*SE*)	Standardized β
Mindfulness		
→ Self-compassion	1.00 (0.05)	0.68***
→ Grit	0.57 (0.09)	0.39***
→ Mind wandering	− 5.83 (0.99)	− 0.31***
Self-compassion		
→ Grit	0.22 (0.06)	0.22***
→ Mind wandering	− 4.02 (0.66)	− 0.32***
Grit		
→ Mind wandering	− 3.05 (0.56)	− 0.24***
Hours of meditation per week^†^		
→ Self-compassion	0.09 (0.04)	0.10*
→ Grit	0.01 (0.04)	0.01
→ Mind wandering	1.52 (0.45)	0.13**
←→ Mindfulness	0.08 (0.03)	0.18**
Age		
→ Self-compassion	0.005 (0.003)	0.06
→ Grit	0.01 (0.003)	0.10*
→ Mind wandering	− 0.05 (0.04)	− 0.06
←→ Mindfulness	0.96 (0.28)	0.18***
Gender (0 = male; 1 = female)		
→ Self-compassion	0.01 (0.06)	0.01
→ Grit	0.06 (0.07)	0.04
→ Mind wandering	− 0.56 (0.70)	− 0.03
←→ Mindfulness	− 0.02 (0.01)	− 0.06
Income		
→ Self-compassion	0.03 (0.01)	0.09*
→ Grit	0.02 (0.01)	0.08*
→ Mind wandering	0.03 (0.12)	0.01
←→ Mindfulness	0.20 (0.08)	0.14**
Education		
→ Self-compassion	0.01 (0.02)	0.02
→ Grit	0.04 (0.02)	0.08
→ Mind wandering	0.05 (0.22)	0.01
←→ Mindfulness	0.01 (0.04)	0.01

Participants’ education ranged from 1 to 6: 1 = Some high school; 2 = High school/College graduate; 3 = Technical/Vocational training; 4 = Associate degree; 5 = University degree; 6 = Postgraduate degree. Monthly household income ranged from 1 to 12: 1 = £0–5000; 2 = £5001–15,000; 3 = £15,001–25,000; 4 = £25,001–35,000; 5 = £35,001–45,000; 6 = £45,001–55,000; 7 = £55,001–65,000; 8 = £65,001–75,000; 9 = £75,001–85,000; 10 = £85,001–95,000; 11 = £95,001–105,000; 12 =  > £105,001.

*p < 0.05, **p < 0.01, ***p < 0.001.

^†^Participants’ hours of meditation per week was ln-transformed to minimize the skewness of the data for analyses.

Based on the above findings, the indirect effects between mindfulness and mind wandering through self-compassion and grit were investigated. Using 10,000 bootstrap samples with replacement, the 95% confidence interval (CI) indicated that the overall standardized indirect effect between mindfulness and mind wandering did not include a zero [CI (− 0.43, − 0.27); β =  − 0.35, *p* < 0.001]. In addition, the specific standardized indirect effect via self-compassion [CI (− 0.30, − 0.14); β =  − 0.22, *p* < 0.001] and grit [CI (− 0.15, − 0.06); β =  − 0.04, *p* = 0.003], respectively, also did not include zeros. Therefore, both self-compassion and grit mediated the relation between mindfulness and mind wandering. Given that the association between mindfulness and mind wandering remained significant (β =  − 0.31, *p* < 0.001) after the inclusion of the mediators, self-compassion and grit partially mediated the relation between mindfulness and mind wandering in this sample of meditators.

## Discussion

Grounded in mindfulness-in-meaning theory^20^, self-determination theory^27^, and the optimal performance and health model of grit^39^, this cross-sectional study indicated self-compassion and grit as partial mediators for the relation between mindfulness and mind wandering, after controlling for the effects of gender, age, hours of meditation per week, income, and education in a sample of meditation practitioners residing in the UK. Consistent with recent findings^8,10,11,61^, the study showed mindfulness and mind wandering as opposing constructs. The study further adds to the literature^12,26^ by showing greater self-compassion and grit as potential underlying mechanisms between mindfulness and mind wandering among meditation practitioners. Although the findings serve as an initial step to identify the cross-sectional relations among the variables under study, future work is necessary to disentangle the bidirectional nature of the associations among mindfulness, self-compassion, grit, and mind wandering.

In this study, mindfulness was positively associated with self-compassion among the participating meditators. That is, being mindful of the present moment without judgement is related to people’s kindness towards themselves. It is also related to a greater insight that suffering is impermanent, that it is inherent to humanity, and that it interconnects sentient beings^12,15^. Nevertheless, the cross-sectional nature of this study precludes us from drawing conclusions on the directionality of effects between mindfulness and self-compassion. In addition, although mindfulness-to-meaning theory^20^ suggests that mindfulness can foster skillful actions and prosocial behavior, the strong correlation between mindfulness and self-compassion (see also^24,34^) suggested that mindfulness and self-compassion might have co-arisen, potentially as a result of meditation practice^62^ as our participants were all meditators. In terms of the model findings, the strong correlation between mindfulness and self-compassion might also have led to multicollinearity. A further examination of the findings, however, revealed that the estimates between mindfulness, self-compassion, and other variables were consistent between the zero-order correlations (see Table 2) and the parameter estimates in the path analysis (see Table 3), thereby ruling out potential statistical artifacts associated with multicollinearity. As theoretically overlapping and distinct concepts, Neff et al.^15,17^ argued that mindfulness forms the basis for self-compassion including self-kindness and common humanity. Nevertheless, the 12-item Self-Compassion Scale Short Form (SCS-SF^54^) included two items of mindfulness (i.e., “When something painful happens I try to take a balanced view of the situation” and “When something upsets me I try to keep my emotions in balance”), which might have accounted for the strong correlation. Future studies should clarify and refine the theoretical nuances between mindfulness and self-compassion, and potentially map the respective measure on each construct. Longitudinal studies and randomized controlled trials are also necessary to further delineate the nuanced processes between meditation, mindfulness, and self-compassion.

Supporting self-determination theory^27^, the presenting findings indicated that mindfulness and self-compassion were positively associated with grit. By being more mindful, people have an increased awareness about their conflicting goals and a greater autonomy to prioritize long-term goals over hedonically pleasant activities (see also^26^). By being more compassionate, people also have a greater realization that setbacks are part of the common humanity^15^. With a greater relatedness to others’ setbacks and experience, they are supported to sustain their motivation and commitment in goal pursuit. They are also less likely to distress over their shortcomings and short-term failures, thereby fostering greater grit^30^.

Furthermore, grit was related to a lower level of mind wandering. As such, gritty individuals have better self-regulation^39,42^ in ignoring distracting thoughts to achieve long-term goals. Self-compassion was also negatively related to mind wandering. With a higher level of self-compassion, individuals are more likely to be gentle with themselves, rather than being fixated on interfering thoughts such as self-criticisms, ruminations, or worries^63^. This inverse relation corroborated another study^37^, which indicated that self-compassion was related to lower mind wandering among people who were mildly to severely depressed.

### Limitations and future directions

The present study must be interpreted in light of several limitations. First of all, the cross-sectional data prevented us from drawing conclusions on the temporal sequence among the variables. Future studies should, therefore, use longitudinal data to disentangle the directionality of effects of the mediation model^64^. Second, the present study used one of the many definitions of mindfulness, i.e., the awareness arising from paying attention to the present moment, on purpose, and without judgment^1^. Nevertheless, this definition was tailored to Western audiences^65^ and may not fully capture the breadth and depth of mindfulness. Given that mindfulness has been conceptualized differently by different scholars^38^, its association with the variables under study may vary, depending on the definitions and measures used. As such, the present findings are limited to our operational definition of mindfulness^1^ and the use of the FFMQ^53^. Similarly, the present study used one of the many definitions of mind wandering, i.e., the engagement with task-unrelated and self-generated and task-unrelated thoughts^9^. However, mind wandering may occur unintentionally or intentionally^66^ or spontaneously or deliberately^67^. Therefore, researchers should investigate the nuances between unintentional (spontaneous) and intentional (deliberate) mind wandering and their relations with mindfulness (see also^68^). Third, we were unable to draw conclusions on the causal effects of mindfulness on self-compassion, grit, and mind wandering. As future directions, researchers could conduct experimental studies or interventions to examine the psychological benefits of mindfulness and self-compassion. Fourth, the present study involved a self-report survey, which could lead to common-method bias^69^. Hence, future studies should use a multi-method multi-informant approach to examine the constructs. Fifth, given that hours of meditation per week was skewed, it was ln-transformed for further analyses. As such, the meaning of this covariate was altered and should be interpreted with caution. Sixth, the current study had a wide range of participants, from new meditators with less than a month of practice to seasoned meditators with many years of practice. Although the inclusion of a range of participants could enhance generalizability, future studies should investigate the potential similarities or differences between new and long-term meditators. Seventh, meditation can take different forms, namely focused meditation, loving-kindness meditation, and mantra meditation^70^. To add specificity to the present findings, future studies should examine how different types of meditation is linked to the present variables under study. Eighth, although a sample of meditators were involved in this study, we did not examine the relations between people’s involvement in meditation and the variables under study. Given that meditation is linked to self-compassion^71^ and is common in mind wandering^72^, future studies should examine the effects of both dispositional mindfulness and meditation on long-term psychological functioning. Nineth, in this study, the MEWS^56^ was used to assess participants’ mind wandering. Even though the measure has been validated and used to assess mind wandering in healthy adults and non-clinical samples^73–75^, the MEWS was originally developed for adults with ADHD^56^. Although Mowlem et al.^57^ indicated measurement invariance across individuals with or without ADHD diagnosis, future studies should utilize multiple measures^76^ and different methods, such as naturalistic observation and probing methods^77^, to assess mind wandering. Tenth, the present study was not pre-registered. To demonstrate credibility, future studies should participate in open science and deposit the study designs and procedures in a repository. Given the above limitations, the findings must be interpreted with caution.

## Conclusions

The present study lends initial support to self-compassion and grit as partial mediators between mindfulness and mind wandering among meditators. As an important take-home message, mindfulness is linked to greater compassion towards oneself, greater grit, and better disengagement from task-unrelated thoughts. Given that the present findings are cross-sectional and correlational, the processes through which mindfulness reduces mind wandering merit further longitudinal and experimental investigations.

## Data Availability

The dataset analyzed in this article is not publicly available. Requests to access the dataset should be directed to Rebecca Y. M. Cheung.
